# Effects of highly active antiretroviral therapy on the survival of HIV-infected adult patients in urban slums of Kenya

**DOI:** 10.11604/pamj.2015.20.63.4865

**Published:** 2015-01-22

**Authors:** Samuel Opondo Muhula, Memiah Peter, Biadgilign Sibhatu, Ndirangu Meshack, Kyomuhangi Lennie

**Affiliations:** 1 African Medical and Research Foundation (AMREF) Kenya; 2 University of Maryland, School of Medicine-Institute of HumanVirology,Baltimore; 3 Independent Public Health Research Consultants, Addis Ababa, Ethiopia

**Keywords:** Haart, survival, slums, Kenya

## Abstract

Recent improvements in access to Anti-Retroviral Therapy (ART) have radically reduced hospitalizations and deaths associated with HIV infection in both developed countries and sub-Saharan Africa. Not much is known about survival of patients on ART in slums. The objective of this study was to identify factors associated with mortality among adult patients on ART in resource poor, urban, sub-Saharan African setting. A prospective open cohort study was conducted with adult patients on ART at a clinic in Kibera slums, Nairobi, Kenya. The patients’ enrollment to care was between March 2005 and November 2011. Descriptive statistics were computed and Kaplan-Meier (KM) methods used to estimate survival time while Cox's proportional hazards (CPH) model fitted to determine mortality predictors. A total of 2,011 adult patients were studied, 69% being female. Female gender (p = 0.0016), zidovudine-based regimen patients (p < 0.0001), CD4 count >351 patients (p < 0.0001), WHO stage I patients (p < 0.0001) and “Working” functional status patients recorded better survival probability on ART. In CPH analysis, the hazard of dying was higher in patients on Stavudine-based regimen(hazard ratio (HR) =1.8; 95% CI, 1.5-2.2; p < 0.0001),CD4 count <50 cells/µl (HR = 1.6; 95% CI, 1.5-1.7;p < 0.0001), WHO Stage IV at ART initiation (HR = 1.3; 95% CI, 1.1-1.6; p = 0.016) and bedridden patients (HR = 2.7; 95% CI, 1.7-4.4;p < 0.0001). There was increased mortality among the males, those with advanced Immunosuppression, late WHO stage and bedridden patients. The findings further justify the need to switch patients on Stavudine-based regimen as per the WHO recommendations.

## Introduction

Recent improvements in access to ART have radically reduced hospitalization rates and deaths associated with HIV infection in both developed countries and Sub-Saharan Africa [1, 2]. Roughly an estimated 35.3 million people were living with HIV worldwide as at the end of 2012. The number of people dying of AIDS-related causes fell to 1.6 million in 2012, down from a peak of 2.2 million in the mid-000s [2]. While sub-Saharan Africa is home to only 12.5% of the world's population, it accounts for about 68% of the world's HIV infected population [2]. The focus on urban poor is warranted since globally, there are more people living in urban areas than in rural areas and the United Nations project that the urban population will grow to 9.3 billion by 2050 in the urban areas of the less developed countries, especially Africa and Asia [3].

In recent years evidence has shown of large health inequalities between the urban slums and other residents [4]. Much of this disadvantage is attributed to poor supply of basic services for health attributed to higher HIV/AIDs burden evidenced by high prevalence rate, cases of mortality as a result of HIV/AIDS [5, 6]. In nearly all of sub-Saharan Africa, a large majority of those infected with HIV and live in the slums do not have access to modern HIV care, especially ART, given the informal nature of these settlements which are characterized by inadequate infrastructure to deliver effective health services. Health planning in such slums is a major challenge due to high mobility of the communities and very little information about the burden of diseases in the slums as a result of insufficient data captured [4, 5]. In addition, lack of adequate food and grinding poverty have been cited as reasons for poor adherence to ART whereby patients have sold their medications to meet the most basic human needs [7, 8]. It is in such context that AMREF established ART program in Kibera slums at the AMREF Kibera Health facility. Since the year 2003 the ART program has enrolled more than 8,000 patients on care and treatment. Kibera is one of Africa's largest urban slums situated in the heart of Nairobi and estimated to have a population of 950,000 people, depending on seasons [9].

While many studies have shown improvements in survival on ART [10, 11], not much is known about the survival experience of patients on ART in such marginalized settings. Such information on survival is urgently required to help programs planners to target slum residents on long term treatment strategies and more accurately forecast ART requirements. The same information is urgently required to provide policy makers and programmers with dynamics that suggest subsequent survival and in turn prevent premature mortality as a result of HIV related complications in the slum settings. The objective of this study was to identify factors associated with mortality among adult patients on HAART in resource urban poor setting in sub-Saharan Africa.

## Methods

Kenya has a population of 38.6 million and about 1.19 million adults aged 15 to 64 years living with HIV infection [12]. This study was conducted at the AMREF Community Health Facility situated at the Kibera slums, Nairobi, Kenya. The 2009 Kenya Population and Housing Census reports Kibera′s population as 170,070, contrary to previous estimates of one or two million people living in an area of approximately 2.5 square kilometers, roughly 5 kilometers away from the city center and is administratively organized into 9 villages [13]. The slum, one of the largest in Africa, has an estimated HIV prevalence of 12% [12]. Several demographic, socio-economic, and cultural factors characterize the Kibera region. The slum has developed into a residence for a mix of communities including the Luos (30%), Kikuyus (20%), Kamba (19%), Luhyas (14%), Kalenjins (6%) and others (11%) many of whom have migrated from their rural homes to the city in search of employment. AMREF through funding from CDC has been providing HIV prevention, care, and treatment support to adults and children, TB diagnosis and treatment, and Prevention of Mother to Child Transmission (PMTCT) for the Kibera residents from the AMREF Kibera HC. These services are provided through an integrated program approach comprising the comprehensive package for HIV/AIDS prevention, treatment and care among adults and children. Specific to this study is ART program which has been running since the year 2003. Care services at the AMREF Kibera Community Based Health Facility are provided by a medical officer, 5 clinical officers, 28 nurses, 3 pharmacists, 3 laboratory technologists, 2 nutritionists and 40 community health workers.

### Study design and population

Patients in the ART program at AMREF Kibera Community Based Facility who were enrolled between March 2005 and September 2011 and follow-up done through November 2011 were used in this study. Routinely collected individual patient line data was captured as part of clinical monitoring and used to design a prospective open cohort study. Data was collected by health care workers (HCWs) using patient encounter forms and entered in an Ms Access database. All HIV positive patients, ≥18 years of age who were enrolled for care and treatment in March 2005 and continued visiting the clinic for follow-up visits were included in the study. The patients were followed up till they died as a result of AIDS related complications or got censored as a result of lost to follow-up, stopped on ART, transferred out or end of the follow-up period in November 2011.

### Measurements

Key variables considered in the study included socio-demographic characteristics and clinical parameters. The program provided free care, including antiretroviral drugs, treatment of opportunistic infections and laboratory investigations, with clinical consultations provided by a doctor, clinical officers or nurses. Eligibility for ART was based on the 2010 WHO recommendations [14]. Drug adherence counseling was provided to all patients both prior to and during ART. The program routinely provided co-trimoxazole prophylaxis as treatment to opportunistic infections and often nutritional support for malnourished individuals especially those with a body mass index of below 18.4 and to all HIV-infected children. CD4 cell counts were measured using either automated or manual methods.

All variables studied in this study were obtained from data gathered from patients registered/enrolled on treatment from the March 2005 till September 2011. Socio-demographic characteristics considered were Gender (Male, Female), Patient age at start of ART, Patient level of education (No formal education, Primary level, Secondary level, Post-Secondary level, Other form of education) and Patient marital status (Divorced, Married, Single, Widowed, Other). Clinical characteristics considered included baseline clinical and laboratory information such as number of months patient been on ARV, patient ARV regimen as at end of the study (First line regimen, Second line regimen), whether the patient changed regimen during the treatment period (Yes, No), major regimen in patient drug combination (AZT based combination, TDF based combination, D4T based combination, ABC/EFV based combination), patient baseline CD4 count measured in cells/mm3 ( 351), patient WAB Stage at start of ARVs (Working, Ambulatory, Bedridden), patient WHO staging at start of ARVs ( Stages I, II, III and IV) and whether patient was diagnosed with opportunistic infection at start of ART (Yes, No), and treatment outcomes (status of patient as at end of study period (censured-Lost to Follow Ups Stopped on ART Transfer Out Active as at end of study period; uncensored-dead). These characteristics were reviewed from patients′ cards. The primary outcome measure was patient survival.

### Statistical analysis

Patient data which was entered in Ms Access database was then exported to excel spreadsheet cleaned and checked for validity before exporting to SAS^®^v9.1 for analysis. Coding of the data for analysis was well done including the missing data points. As part of the exploratory and descriptive analysis medians, inter-quartile range, means and standard deviations were computed for numerical variables and proportions for categorical variables. Kaplan-Meier analysis [15] was used to test estimate survival time. Log-rank test was used to test significant differences between various groups. Cox proportional hazards model was used to investigate for predictors to mortality among the factors associated to mortality. The study design involved right censoring of patients with incomplete information or whether the patient did not achieve the event of interest (die) during the follow-up period. All patients who did not die were censored at the time of data analysis.

### Ethical consideration

Ethical clearance was obtained from the Ethical Review Committee which is an Institutional Research Ethics Review Committee chaired by Kenya Medical Research Institute (KEMRI). Patient data recorded in patient encounter forms were filled and kept confidential in lockable cabinets within the clinic. Patient identifiers in the data used in this study were made anonymous to avoid linking the patients to their characteristics.

## Results

### Socio demographic characteristics

A total of 2011 HIV positive adult (> 14 years) patients were studied, 69% being female and 31% male. The patient's median age at start of ART was 37 years (IQR 32-45). Majority of the patients (72%) had primary level education and 52% were married. The outcome of the cohort showed 147 (7%) died as a result of AIDS related complications, 2% lost to follow-up, 2% stopped treatment by a clinical person due to various reasons, 9% transferred out and 79% were still alive. Table 1 below shows a summary of demographic characteristics of the HIV-infected Adult patients (Table 1).

**Table 1:** Demographic details among HIV-Infected Adult Patients in Urban Slums of Kenya, 2014

Patient characteristics	n (%)	Mean survival time (Months)
Total	2011	
Gender		
Male	625 (31%)	58.4
Female	1386 (69%)	47.1
Education		
No formal education	50 (4.8%)	33.4
Primary	745 (71.5%)	60.1
Secondary	170 (16.3%)	29.8
Post- Secondary	50 (4.8%)	35.7
Other	27 (2.6%)	25.1
Missing data	969	
Marital		
Divorced	106 (7.5%)	
Married	741 (52.1%)	55.6
Single	381 (26.8%)	59.2
Widowed	187 (13.2%)	35.7
Other	7 (0.5%)	3
Missing data	589	

### Clinical care characteristics

**Figure 1:**
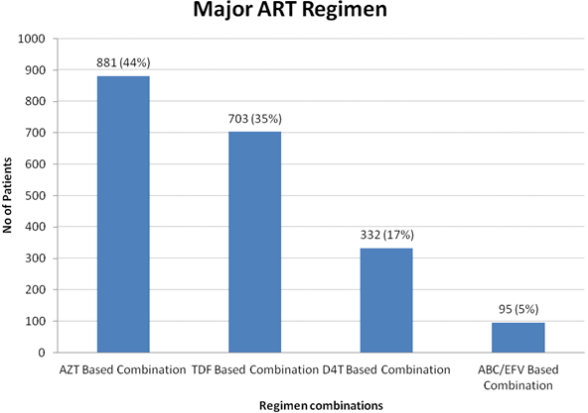
Major ART Regimen among HIV-Infected Adult Patients in Urban Slums of Kenya, 2014

The median baseline CD4 cell count was 210 cells/µl (IQR 106-315, n = 1,852), and 35%, 27%, 24% and 14% were classified as WHO clinical stages I, II, III and IV respectively. The median duration of patients on ART was 22 months (IQR 10-44) before dying or being censored, with 983 (49%) patients staying on treatment for more than 22 months. First line ART regimen most frequently prescribed was Zidovudine-based regimen (44%) followed by Tenofovir-based regimen 35% and Stavudine-based regimen at 17% as shown in(Figure 1) below. Participants who changed regimen during the treatment period were 38% of the total. Below is a summary of clinical details among HIV-infected Adult patients reviewed in the study (Table 2).

**Table 2:** Clinical details among HIV-Infected Adult Patients in Urban Slums of Kenya, 2014

Patient characteristics	n (%)	Mean survival time (Months)
Regimen		
1^st^ line	1919 (95%)	60
2^nd^ line	92 (5%)	16.6
Changed regimen		
Yes	756 (38%)	61.6
No	1255 (62%)	35
Major regimen		
AZT based combination	881 (44%)	56.5
TDF based combination	703 (35%)	61
D4T based combination	332 (17%)	25.7
ABC/EFV based combination	95 (5%)	24.6
CD4 Cell Count Range		
<50	218(12%)	52.4
51-100	220 (12%)	33.9
101-200	437 (24%)	40.1
201-350	629 (34%)	48.5
>351	348 (19%)	57.4
WAB stage		
Working	1878 (95.4%)	61.2
Ambulatory	83 (4.2%)	30
Bedridden	7 (0.4%)	3
Missing data	43	
WHO ART		
Stage 1	499 (26.8%)	62.6
Stage 2	266 (14.3%)	30.2
Stage 3	653 (35%)	36
Stage 4	446 (24%)	32.5
Missing data	147	
Opportunistic Infection (OI)		
Yes	335 (17%)	37.8
No	1676 (83%)	60.6

### Factors associated with mortality

The probability of remaining alive and on treatment after 22 months of follow-up was 0.89 (95% CI 0.88-0.91) for male patients and 0.94 (95% CI 0.93-0.94) for female patients. In bivariate analysis, female gender (p = 0.0016), zidovudine-based regimen patients (p351 patients (pFigure 2 and Figure 3) below show the Kaplan-Meier Curves was estimating survival probabilities of patients by CD4 Range and WHO staging respectively. In multivariate analysis using Cox proportional hazards model, the hazard of dying was higher in patients on Stavudine-based regimen (hazard ratio (HR) =1.8; 95% CI, 1.5-2.2; pTable 3 below is a summary of Hazard ratios of mortality analysis of Maximum Likelihood estimates among HIV-Infected Adult Patients in Urban Slums of Kenya, 2014(Table 3)

**Figure 2:**
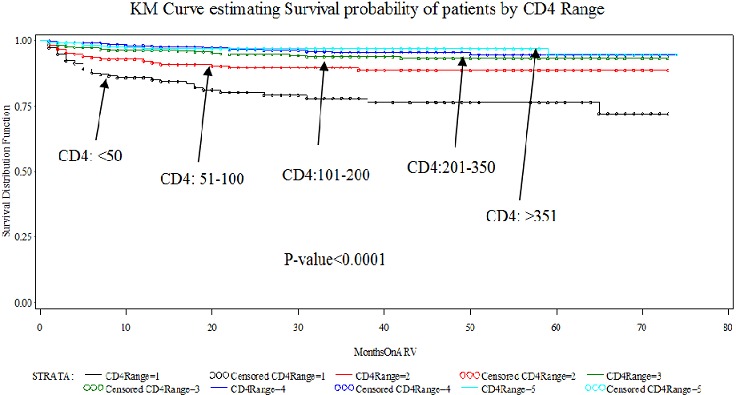
Kaplan-Meier Curve estimating Survival probability of patients by CD4 Range

**Figure 3:**
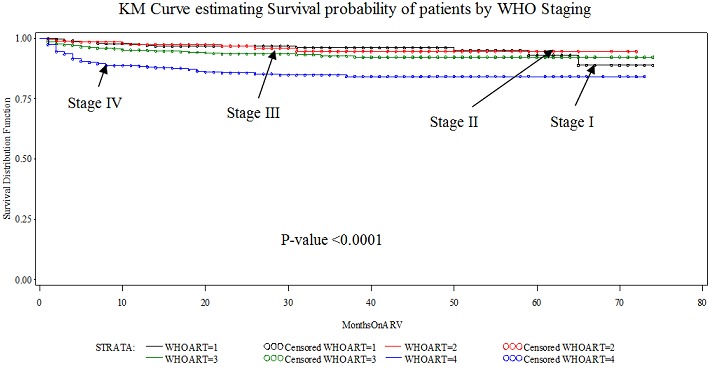
Kaplan-Meier Curve estimating Survival probability of patients by WHO Staging

**Table 3:** Hazard ratios of mortality analysis of Maximum Likelihood estimates among HIV-Infected Adult Patients in Urban Slums of Kenya, 2014

Factor	Pr > ChiSq	Hazard Ratio	95% Hazard Ratio Confidence Limits
CD4 Cell count	<.0001	0.6	(0.5 0.7)
Patients changing regimen	0.0044	1.9	(1.2 2.9)
Major ART Regimen	<.0001	1.8	(1.5 2.2)
Patients Functional status	<.0001	2.7	(1.7 4.4)
WHO Staging at start of ART	0.0096	1.3	(1.1 1.6)

## Discussion

This study confirms some of the findings in the literature with regard to ART treatment in patients and yet also provides some contrasting evidence in some aspects. Our study confirms the generally held view that low CD4 count in ART patients is a predictor for the progression to death. Similar results have been demonstrated with other studies. In a big cohort of collaborative study from Europe and North America show that baseline CD4 cell count was strongly associated with the probability of progression to AIDS or death [16]. Baseline CD4 cell count (adjusted hazard ratio of 0.21 (95% CI 0.17-0.27)comparing ≥200 with 17]. Death was associated with lower CD4 (adjusted hazard ratio (HR) for CD4 = 200 vs CD4 = 50 was 0.58; 95% CI: 0.40-0.85) [18], baseline CD4 counts were associated with mortality [19]. In an Ethiopian study on determinates of survival in adult HIV patients on ART in Oromiyaa, there is no association found between CD4 count and survival [20]. In contrary, in eastern Ethiopia, less than 200 CD4 cells/m were highly associated with death [21].

Relative to a baseline CD4 cell count of less than 50 cells/ml, the risk of mortality was 0.75, 0.60, 0.43, and 0.41 for those with baseline CD4 cell counts of 50-99, 100-149, 150-249, and 250 cells/µl. [22] and significant risk factors associated with mortality baseline CD4 cell count under 50 cells/mul [23] and CD4 counts were independent predictors of mortality in a study in Far-North Province of Cameroon [24]. Similarly finding has been reported in sub-Saharan Africa in that there was strong evidence for a decline in excess risk with increasing baseline CD4 count: patients starting with a CD4 count of 200 cells/ml or more experienced an 81% reduction in risk over 2 years compared to patients that started with a CD4 count of less than 25cells/ml(eHR 0.19) [25]. In another study, death was associated with lower CD4 (adjusted HR for CD4 = 200 vs. CD4 = 50 was 0.58; 95% CI: 0.40 to 0.85) and the mortality rate was higher (p 26].

In our study bedridden patients was associated with higher mortality as compared to ambulatory. Another study in Ethiopia found patient functional status to be associated with mortality in the bivariate analysis [19]. Similar findings have been documented elsewhere. In eastern Ethiopia study, bedridden functional status at baseline had a higher risk of death among the retrospective cohort [21]. Patients in ambulatory functional status and bedridden are at increased hazard rate of death by 2.7 and 2.382 times than patients in working functional status respectively [27]. This might be due to the fact that bedridden patients have been long in bed so that there clinical characteristics are deteriorating and the disease progression has implicated here with low CD4 count.

In this study another determinant of mortality was WHO clinical stages. In Ethiopian study, a statistically significant difference in survival probability was observed among WHO clinical stages, ranging from 0.96 for clinical stage I/II to 0.74 for stage IV, p20]. In Malawi study, significant risk factors associated with mortality were WHO stage IV disease [23] and in Far-North Province, Cameroon, clinical stage at enrollment were independent predictors of mortality [24]. In Brinkhof and colleagues study, the excess risk was reduced by 72% (eHR =0.28) over the 2 year in patients starting with less advanced disease (WHO stage I/II) compared to patients starting with advanced disease (WHO stage III/IV) [25] and death was associated with clinical AIDS (hazard ratio = 3.1; 95% CI: 2.1 to 4.5) [18]. Baseline WHO stage IV was the strongest predictor of death in the first 4 weeks (HR = 9.2) [28]. Stage 4 disease was the strongest risk factor for mortality in south Africa [29] and baseline WHO staging III and IV (AHR = 2.16, 95% CI: 1.10 to 4.25) in Ethiopia [27]. Advanced clinical condition (WHO stages III and IV) were associated independently with increased mortality in south-east Uganda and eastern Ethiopia [21, 30]. There is a clinical significant attribute to advance disease stage for the mortality of patients.

In our study, treatment regimen has been associated with mortality. In sub-Saharan African study, there was little evidence for an association between excess mortality and treatment regimen [25] and compared with EFV, the adjusted HR for all-cause mortality for NVP was 2.28 during the first 6 months, which decreased to 1.31 after 6 month. The mortality rates did not vary (p >0.05) between the 3 ART regimens: 8.8% (n = 184/2083) in NRTI (1a regimen), 8.9% (n = 7/78), and 8% (n = 2/26) in regimen 2(3rd NRTI) [26]. This study has limitations and strengths. In some of the patients misclassification bias were there to differentiate the lost and dead cases for censoring, difficult to ascertain the cause of the death in some patients, and incompleteness of some of the variables like the CD4 measurements and selection bias were anticipated. Despite this, the study address the marginalized community found in slum of the Kenya, relatively a large prospective cohort study that demonstrated in sub-Saharan setting and large sample size gives a clear picture on the factor that affect the mortality in developing countries.

## Conclusion

In conclusion, there was increased mortality among male patients, patients with advanced Immunosuppression, patients on Stavudine-based regimen, those in late World Health Organization Stage 4 at ART initiation and those bedridden patients. The findings further justify the need to switch patients on Stavudine-based regimen to Tenofovir as per the WHO recommendations.
